# Active Trachoma Cases in the Solomon Islands Have Varied Polymicrobial Community Structures but Do Not Associate with Individual Non-Chlamydial Pathogens of the Eye

**DOI:** 10.3389/fmed.2017.00251

**Published:** 2018-01-23

**Authors:** Robert M. R. Butcher, Oliver Sokana, Kelvin Jack, Eric Kalae, Leslie Sui, Charles Russell, Joanna Houghton, Christine Palmer, Martin J. Holland, Richard T. Le Mesurier, Anthony W. Solomon, David C. W. Mabey, Chrissy h. Roberts

**Affiliations:** ^1^Clinical Research Department, London School of Hygiene & Tropical Medicine, London, United Kingdom; ^2^Eye Department, Ministry of Health and Medical Services, Honiara, Solomon Islands; ^3^Primary Care Department, Lata Hospital, Santa Cruz Island, Solomon Islands; ^4^Bellona Rural Health Centre, Bellona, Solomon Islands; ^5^The Fred Hollows Foundation, Carlton, VIC, Australia

**Keywords:** droplet digital PCR, Solomon Islands, 16S rRNA gene sequencing, trachoma, non-chlamydial infections

## Abstract

**Background:**

Several non-chlamydial microbial pathogens are associated with clinical signs of active trachoma in trachoma-endemic communities with a low prevalence of ocular *Chlamydia trachomatis* (*Ct*) infection. In the Solomon Islands, the prevalence of *Ct* among children is low despite the prevalence of active trachoma being moderate. Therefore, we set out to investigate whether active trachoma was associated with a common non-chlamydial infection or with a dominant polymicrobial community dysbiosis in the Solomon Islands.

**Methods:**

We studied DNA from conjunctival swabs collected from 257 Solomon Islanders with active trachoma and matched controls. Droplet digital PCR was used to test for pathogens suspected to be able to induce follicular conjunctivitis. Polymicrobial community diversity and composition were studied by sequencing of hypervariable regions of the 16S ribosomal ribonucleic acid gene in a subset of 54 cases and 53 controls.

**Results:**

Although *Ct* was associated with active trachoma, the number of infections was low (cases, 3.9%; controls, 0.4%). Estimated prevalence (cases and controls, respectively) of each non-chlamydial infection was as follows: *Staphylococcus aureus*: 1.9 and 1.9%, Adenoviridae: 1.2 and 1.2%, coagulase-negative *Staphylococcus*: 5.8 and 4.3%, *Haemophilus influenzae*: 7.4 and 11.7%, *Moraxella catarrhalis*: 2.3 and 4.7%, and *Streptococcus pneumoniae*: 7.0 and 6.2%. There was no statistically significant association between the clinical signs of trachoma and the presence or load of any of the non-*Ct* infections that were assayed. Interindividual variations in the conjunctival microbiome were characterized by differences in the levels of *Corynebacterium, Propionibacterium, Helicobacter*, and *Paracoccus*, but diversity and relative abundance of these specific genera did not differ significantly between cases and controls.

**Discussion:**

It is unlikely that the prevalent trachoma-like follicular conjunctivitis in this region of the Solomon Islands has a dominant bacterial etiology. Before implementing community-wide azithromycin distribution for trachoma, policy makers should consider that clinical signs of trachoma can be observed in the absence of any detectable azithromycin-susceptible organism.

## Introduction

Trachoma, caused by *Chlamydia trachomatis* (*Ct*), is the leading infectious cause of preventable blindness (1) and is targeted for elimination as a public health problem by 2020 through the SAFE strategy (Surgery, Antibiotics, Facial cleanliness and Environmental improvement). The decision to implement community-wide trachoma control interventions, which include mass drug administration (MDA) with azithromycin, is based on population prevalence estimates of one clinical sign of active trachoma [trachomatous inflammation–follicular (TF)] in the 1- to 9-year-old age group (2). In the Solomon Islands, the prevalence of TF is sufficient to warrant MDA, but the prevalence of trachomatous trichiasis (TT) suggests that trachoma may not pose a significant public health problem. A recent trachoma survey in a treatment-naive population of the Solomon Islands estimated 26% of 1 to 9 year olds to have TF, but, surprisingly, conjunctival *Ct* infection was detected in only 1.3% of that age group (3). While *Ct* infection is not always detectable in TF cases (4, 5), infection prevalence in the Solomon Islands is far lower than is seen in other countries with similar TF prevalence (3).

*Chlamydia trachomatis* is not the only pathogen to associate with signs of TF. A number of differential diagnoses for follicular conjunctivitis are described (6), some of which are rare and distinguishable by patient history (such as Parinaud’s oculoglandular syndrome). Other causes of conjunctivitis, which can present with follicles, are *Streptococcus pneumoniae, Haemophilus influenzae* (7), and adenovirus (8). Non-ocular *Chlamydia* serotypes (9) and non-*Ct* species (10) have also been suggested as possible causes of TF. A number of studies have examined conjunctival microbiology in trachoma-endemic areas. A greater diversity of pathogens can be cultured from the conjunctivae of people with TF compared to counterparts without TF (7). Among bacterial species isolated during a study in Tanzanian children, *S. pneumoniae, H. influenzae* B, and *H. influenzae* non-type B associated more strongly than *Ct* with clinical signs of active trachoma (7). In The Gambia, *S. pneumoniae* and *H. influenzae* type B infection correlated closely with signs of active trachoma in communities that had previously received MDA, whereas *Moraxella catarrhalis* and *Staphylococcus aureus* did not (11). Common non-chlamydial pathogens have associated with trachomatous scarring (TS) (12) and recurrence of TT after surgery (13) in some, but not all (13, 14), studies.

Although traditionally thought to be “sterile”, several studies have now described polymicrobial communities colonizing the conjunctival epithelium (15, 16). *Staphylococcus, Streptococcus, Haemophilus*, and *Moraxella* genera can be readily cultured from swabs taken from the inferior fornix (7, 11, 12). Attempts to detect bacteria from the conjunctiva have shown community diversity and composition to vary significantly between individuals. It is unclear whether a “core” microbiota persists at the conjunctiva, but profiles closely related to that of the skin have been reported (17). *Corynebacterium, Propionibacterium, Staphylococcus*, and *Streptococcus* have been consistently dominant, whereas other genera such as *Acinetobacter, Brevundimonas, Pseudomonas, Bradyrhizobium, Sphingomonas, Bacillus, Simonsiella*, and *Elizabethkingia* have been identified more sporadically (18–20). Conjunctival polymicrobial community composition is known to vary with age and season (21) and appears to be responsive to external stimuli such as regular contact lens wear (22). The conjunctival microbiome varies significantly between people with TS and those without; however, significant associations between microbiome and follicular conjunctival inflammation or microbiome and other diseases have yet to be described (21, 23).

We hypothesized that clinical signs of active trachoma in the Solomon Islands where ocular *Ct* is uncommon could be explained by a common non-chlamydial infection or by a dominant polymicrobial community dysbiosis.

## Materials and Methods

### Study Ethics

A parent or guardian gave written informed consent for each child to take part in the survey in accordance with the Declaration of Helsinki. The protocol was approved by the London School of Hygiene & Tropical Medicine (6319/6360) and the Solomon Islands National Health Research (HRC13/18) Ethics Committees.

### Study Population

The samples tested during this study were a subset of specimens from a population-based trachoma prevalence survey of 3,674 people (1,135 children aged 1–9 years) in 32 clusters from Temotu and Rennell & Bellona Provinces, Solomon Islands; data from that survey are presented elsewhere (3). At the time of the survey, no trachoma interventions had been implemented in the Solomon Islands. Sterile swabs were passed three times over the right conjunctiva of every child before storage in 300 µL RNALater^®^ (Life Technologies). Swabs were kept cool in the field for up to 48 h then frozen until processing (3). Inclusion criteria for cases (*n* = 257) were age 1–9 years, detectable human DNA in the conjunctival swab, and a field grade of TF or trachomatous inflammation—intense (TI) in the right eye. An equal number (*n* = 257) of age-, gender- and island-matched controls without TF or TI were selected using the “e1071” R package. A random subset of cases (*n* = 54) and controls (*n* = 53) were further characterized using V1–V3 16S ribosomal RNA (rRNA) gene sequencing. Field controls (clean swabs passed within 20 cm of seated participants, without touching them, and then treated identically to subjects’ specimens), extraction controls (extraction carried out in the absence of a specimen and PCR carried out on the eluate), and PCR controls (PCR carried out in the absence of any added material) were used to determine background levels of microbial contamination in the 16S rRNA gene sequencing protocol.

### Droplet Digital PCR

DNA was extracted from conjunctival swabs using the Qiagen AllPrep DNA/RNA kit. We chose not to use mechanical lysis on these low biomass specimens due to the reported lower yields compared to chemical lysis (24). All specimens have previously been tested for *Ct* and *Homo sapiens* ribonuclease P protein subunit p30 (acting as an endogenous control) using a validated assay (25); the community prevalence of ocular *Ct* infection is described elsewhere (3).

Duplex ddPCR assays were developed for (1) *Adenoviridae* (26) and *S. pneumoniae* (2, 27), *H. influenzae* (28) and *M. catarrhalis* (29, 30), and *S. aureus* and coagulase-negative *Staphylococcus* (31, 32) based on the published assays. Each assay contained 10 µL 2× ddPCR Supermix for probes (Bio-rad, Hemel Hempstead, UK), primers and probes at custom concentrations (Table 1), and 8 µL of template DNA. Thermal cycling was performed for 10 mins at 95°C, 40 cycles of 15 seconds at 95°C then 1 min at 60°C, followed by 12 minutes at 98°C. A positive result for a clinical specimen was defined as >95% confidence of non-zero target load, as described previously (25). Assay performance was assessed by repeat testing of PCR product dilution series’ and cultured pathogen material before rolling out to clinical samples. The reproducibility, linearity, and limits of detection of each assay were considered appropriate (Table S1 in Supplementary Material) and were also similar to published performance of the *Ct* assay used (25).

**Table 1:** Oligonucleotides used in this study, with assay concentrations derived from *in vitro* optimization.

Organism/target	Oligo	Sequence (5′-3′)	Concentration in final assay (nM)	Amplicon size	Reference
*Chlamydia trachomatis*/plasmid open reading frame 2 (*pORF2*)	F	CAG CTT GTA GTC CTG CTT GAG AGA	900	109	(3, 25, 33)
	R	CAA GAG TAC ATC GGT CAA CGA AGA	900		
	Probe	[FAM] CCC CAC CAT TTT TCC GGA GCG A [BHQ1]	200		

*Staphylococcus aureus*/Staphylococcal protein A (*SpA*)	F	CAG CAA ACC ATG CAG ATG CTA	900	101	(31, 32)
	R	CGC TAA TGA TAA TCC ACC AAA TAC A	900		
	Probe	[VIC] TCA AGC ATT ACC AGA AAC [MGBNFQ]	250		

Coagulase-negative *Staphylococcus*/translation elongation factor (*tuf*)	F	TAT CCA CGA AAC TTC TAA AAC AAC TGT TAC T	450	204	(32)
	R	TCT TTA GAT AAT ACG TAT ACT TCA GCT TTG AAT TT	450		
	Probe	[FAM] TAT TAG ACT ACG CTG AAG CTG GTG ACA ACA T [BHQ1]	125		

*Streptococcus pneumoniae*/*N*-acetylmuramoyl-l-alanine amidase (*lytA*)	F	ACG CAA TCT AGC AGA TGA AGC A	500	75	(27)
	R	TCG TGC GTT TTA ATT CCA GCT	500		
	Probe	[FAM] GCC GAA AAC GCT TGA TAC AGG GAG [BHQ1]	150		

*Haemophilus influenzae*/l-fuculokinase(*fucK*)^b^	F	ATG GCG GGA ACA TCA ATG A	900	102	(28)
	R	ACG CAT AGG AGG GAA ATG GTT	900		
	Probe^a^	[FAM] CGg TAa TTg GGa TCc AT [BHQ1]	125		

*Adenoviridae*/capsid assembly protein (hexon)	F	GCC ACG GTG GGG TTT CTA AAC TT	500	132	(26)
	R	GCC CCA GTG GTC TTA CAT GCA CAT C	500		
	Probe	[HEX] TGC ACC AGA CCC GGG CTC AGG TAC TCC GA [BHQ1]	125		

*Moraxella catarrhalis*/outer membrane protein (*copB*)	F	GTG AGT GCC GCT TTT ACA ACC	900	72	(29, 30)
	R	TGT ATC GCC TGC CAA GAC AA	900		
	Probe	[HEX] TGC TTT TGC AGC TGT TAG CCA GCC TAA [BHQ1]	250		

*^a^Lower case symbols denote locked nucleic acid bases*.

*^b^These primers do not differentiate between encapsulated and non-capsulated subtype*.

### 16S rRNA Gene Sequencing

An approximately 530-bp region of the 16S rRNA gene (variable regions 1–3) was amplified using forward (modified 27F; 5′-[adaptor]-AGAGTTTGGATCCTGGCTCAG-3′) and custom barcoded reverse primers (534R; 5′-[adaptor]-[barcode]-AGTCAGTCAGCCATTACCGCGGCTGCTGG-3′). Each 15 µL reaction contained 7.5 µL 2× Phusion High Fidelity Master Mix (New England Biosciences, MA, USA), 0.45 µL DMSO, 0.1 µM primers, and 5.55 µL DNA. The thermal cycling conditions were as follows: 30 seconds at 98°C followed by 31 cycles of 10 seconds at 98°C then 30 seconds at 62°C followed by 7 minutes at 72°C. Amplicons were cleaned using 0.6 v/v AMPure XP beads (Beckman Coulter, CA, USA), quantified using Qubit (Thermo Fisher Scientific, MA, USA), and then pooled at equimolar concentrations. The 4-nM sequencing library was mixed 0.75 v/v with a 4 nM Phi-X control library (Illumina, CA, USA). 3 µL of 100 µM custom read primers were mixed into wells 12 (Read 1; CTACACTATGGTAATTGTAGAGTTTGGATCCTGGCTCAG), 13 (Index; CCAGCAGCCGCGGTAATGGCTGACTGACT), and 14 (Read 2; AGTCAGTCAGCCATTACCGCGGCTGCTGG) of the MiSeq reagents cartridge before 2 × 300 bp paired-end sequencing with the 600-cycle MiSeq v3 sequencing reagent kit on the MiSeq platform using a standard Illumina protocol. 96 uniquely barcoded specimens were run in multiplex on each MiSeq run.

### Data Analysis

ddPCR data were analyzed using R version 3.2.2 (34). Binomial univariate regression was used to test the relationship between each individual infection and active trachoma. The most accurate final multivariate model, determined by the lowest Akaike Information Criterion value, was determined by stepwise removal of variables from a binomial multivariate regression model incorporating all tested pathogens. The chance of the *Ct* association occurring due to chance was assessed by counting the number of significant results obtained from randomly reordering the *Ct* data 1,000 times.

Raw 16S amplicon sequences were directly assigned to genera using Illumina BaseSpace “16S Metagenomics” app version 1.0.1.0, which uses algorithms from Wang et al. (35). Two clinical specimens yielded fewer than 1,000 reads in total and were removed from the analysis. Amplicons were sequenced from six no-template control (NTC) specimens (two “field” controls, two “extraction” controls, and two “PCR” controls, defined above) to identify any contaminants endogenous to the collection, PCR, or sequencing process. Any genera represented by more than 600 reads in the 6 combined NTCs (average 100 reads per NTC) were eliminated from the clinical specimen analysis. This resulted in the removal of 21 genera, which accounted for 95.1% of the reads from NTCs and 88.3% of the reads from clinical specimens. The genera removed included common contaminants of reagent kits (36) such as *Pelomonas*, as well as some previously described conjunctival microbiota constituents such as *Brevundimonas, Staphylococcus*, and *Streptococcus* (18, 21).

Between-group differences in the Shannon Diversity Index (a measure of diversity, which increases with increasing species abundance and evenness) and Inverse Simpson Index (another measure of diversity where 1 is infinite diversity and 0 is no diversity) were compared using a *t*-test. Discriminant analysis of principal components, a multivariate clustering method to analyze highly dimensional datasets, was performed using the “adegenet” package in R (37), and cross-validation was used to determine the optimal number of principal components (PCs) to include in a discriminant function aimed at separating cases from controls on the basis of their polymicrobial community structures.

## Results

### Specimen Set Demographics

A total of 257 cases and 257 controls (*n* = 514) were tested. All 257 cases had TF, and one also had TI. Case status was defined by clinical signs in the right eye, but 236/257 (91.8%) and 8/257 (3.1%) of the cases and controls, respectively, had TF in the left eye. Both case and control groups were 38% female. Mean age was 5.6 years (cases) and 5.5 years (controls; Student’s *t*-test *p* = 0.76). There was no significant difference between cases and controls in terms of the clusters represented (Kolmogorov–Smirnov test *p* = 0.97), and all 32 clusters were represented. The case specimens had higher loads of human DNA than the controls (18,030 versus 9,354 copies/swab; *p* = 0.00003). There were no significant differences in age, gender, or location within the subset selected for 16S-amplicon sequencing.

### Quantitative PCR Tests for Ocular Pathogens

**Figure 1:**
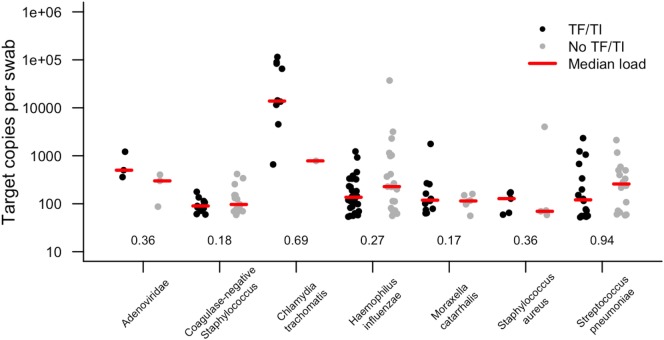
Target copies per swab of each pathogen identified in conjunctival swabs collected from children with and without active trachoma in the Solomon Islands. Numbers show *p* values for logistic regression comparison between active trachoma case and control groups for each pathogen. None of the differences observed were statistically significant. TF, trachomatous inflammation–follicular; TI, trachomatous inflammation—intense.

In this study, 19.8% of children had evidence of infection with at least one of the targeted organisms (Table 2). We considered the prevalence of *Ct* in this sample set to be too low (96.1% of TF cases were *Ct* infection negative) to account for the level of active disease. The prevalence of both *Adenoviridae* (1.2%) and *S. aureus* (1.9%) were very low. There was no association between active trachoma and infection with *H. influenzae, S. pneumoniae, S. aureus*, coagulase-negative *Staphylococcus*, or *M. catarrhalis. Ct* infection was associated with active trachoma (logistic regression *p* = 0.026; odds ratio, 10.4). The association between *Ct* and active trachoma was still significant in a multivariate analysis (*p* = 0.025). The permuted *p* value for the association between *Ct* and active trachoma was highly significant (*p* = 0.001). Active disease was neither associated with infection with at least one pathogen (any pathogen, *p* = 0.185) nor the number of concurrent infections in the same eye (number of concurrent pathogens, *p* = 0.207). Infection loads of those who tested positive are shown in Figure 1. Despite some numerical differences between groups, none of the differences were statistically significant.

**Table 2:** Cases of infection in specimens from children with and without active disease.

Pathogen	ddPCR result	No TF/TI (%; *n* = 257)	TF/TI (%; *n* = 257)	Total (%; *n* = 514)	Univariate odds ratio (95% CI)	Univariate (*p* value)	Multivariate odds ratio (95% CI)	Multivariate (*p* value)
*Adenoviridae*	Positive	3 (1.2)	3 (1.2)	6 (1.2)	1.00 (0.67–1.50)	1.000	–	–
	Negative	254 (98.8)	254 (98.8)	508 (98.8)				

*Chlamydia trachomatis*^a^	Positive	1 (0.4)	10 (3.9)	11 (2.1)	10.36 (1.96–190.92)	**0.026**	10.64 (2.15–196.00)	**0.025**
	Negative	256 (99.6)	247 (96.1)	503 (97.9)				

Coagulase-negative *Staphylococcus*	Positive	15 (5.8)	11 (4.3)	26 (5.1)	0.72 (0.32–1.59)	0.423	–	–
	Negative	242 (94.2)	246 (95.7)	488 (94.9)				

*Haemophilus influenzae*	Positive	19 (7.4)	30 (11.7)	49 (9.5)	1.66 (0.91–3.07)	0.101	–	–
	Negative	238 (92.6)	227 (88.3)	465 (90.4)				

*Moraxella catarrhalis*	Positive	6 (2.3)	12 (4.7)	18 (3.5)	2.05 (0.78–5.96)	0.158	2.13 (0.81–6.20)	0.137
	Negative	251 (97.7)	245 (95.3)	496 (96.5)				

*Staphylococcus aureus*	Positive	5 (1.9)	5 (1.9)	10 (1.9)	1.00 (0.28–3.64)	1.000	–	–
	Negative	252 (98.1)	252 (98.1)	504 (98.1)				

*Streptococcus pneumoniae*	Positive	18 (7.0)	16 (6.2)	34 (6.6)	0.88 (0.43–1.77)	0.723	–	–
	Negative	239 (93.0)	241 (93.8)	480 (93.4)				

Any pathogen	Positive	45 (17.5)	57 (22.2)	102 (19.8)	0.75 (0.48–1.15)	0.185	Not included	
	Negative	212 (82.4)	200 (77.8)	412 (80.2)				

Different pathogen species in the same eye	0	212 (82.4)	200 (77.8)	412 (80.2)	1.21 (0.90–1.63)	0.207	Not included	
	1	28 (10.9)	35 (13.6)	63 (12.3)				
	>1	17 (6.6)	22 (8.6)	39 (7.6)				

*The relationship of those infections to trachoma has been tested with univariate logistic regression, and then stepwise removal of variables from multivariate regression model was used to determine the final multivariate regression model that provided the best fit for the data*.

*^a^Data taken from the study by Butcher et al. (3)*. *Bold values indicate significant p-values < 0.05*.

### 16S rRNA Gene Sequencing

The total number of genera identified across all clinical specimens and NTCs was 659; however, 125/659 (19%) had a cumulative total of 10 reads or fewer. The median number of reads per clinical specimen was 55,104 (interquartile range [IQR]: 40,705–89,541), and the median percentage of reads mapped to genus level was 51.9%. The median number of genera identified was 151 (IQR: 117–212) per clinical specimen. After the removal of presumed contaminants, approximately 40% of cleaned reads were assigned to genera that each constituted less than 1% of the total polymicrobial community. 34 genera were represented by at least 1% of remaining reads in clinical samples.

The diversity of bacterial communities was in general low; the mean Inverse Simpsons Diversity index over all specimens was 0.061 (IQR: 0.046–0.092). The median Inverse Simpson’s Diversity index in cases was 0.061 (IQR: 0.048–0.095) versus controls 0.060 (IQR: 0.044–0.078) (Student’s *t*-test *p* = 0.56). The median Shannon Diversity Index was 3.38 in cases and 3.37 in controls (Student’s *t*-test *p* = 0.96). There was no significant difference in alpha-diversity between cases and controls by either measure of diversity. In cases of active trachoma, the most dominant bacterial genera were *Corynebacterium* (12.0% of total reads), *Propionibacterium* (6.2%), and *Helicobacter* (4.8%). In controls, the most dominant genera were *Corynebacterium* (13.9%), *Paracoccus* (5.2%), *Propionibacterium* (4.7%), and *Neisseria* (4.1%; Figure S1 in Supplementary Material). The genus level membership of the bacterial community varied between cases and controls. Of 21 genera represented by at least 1% of reads in specimens from those with active trachoma, 10 were not found in controls, including *Helicobacter, Mesoplasma, Brachybacterium*, and *Haemophilus*. Of 23 genera found in controls, 12 were not found in cases, including *Neisseria, Prevotella, Rhodococcus*, and *Porphyromonas*.

**Figure 2:**
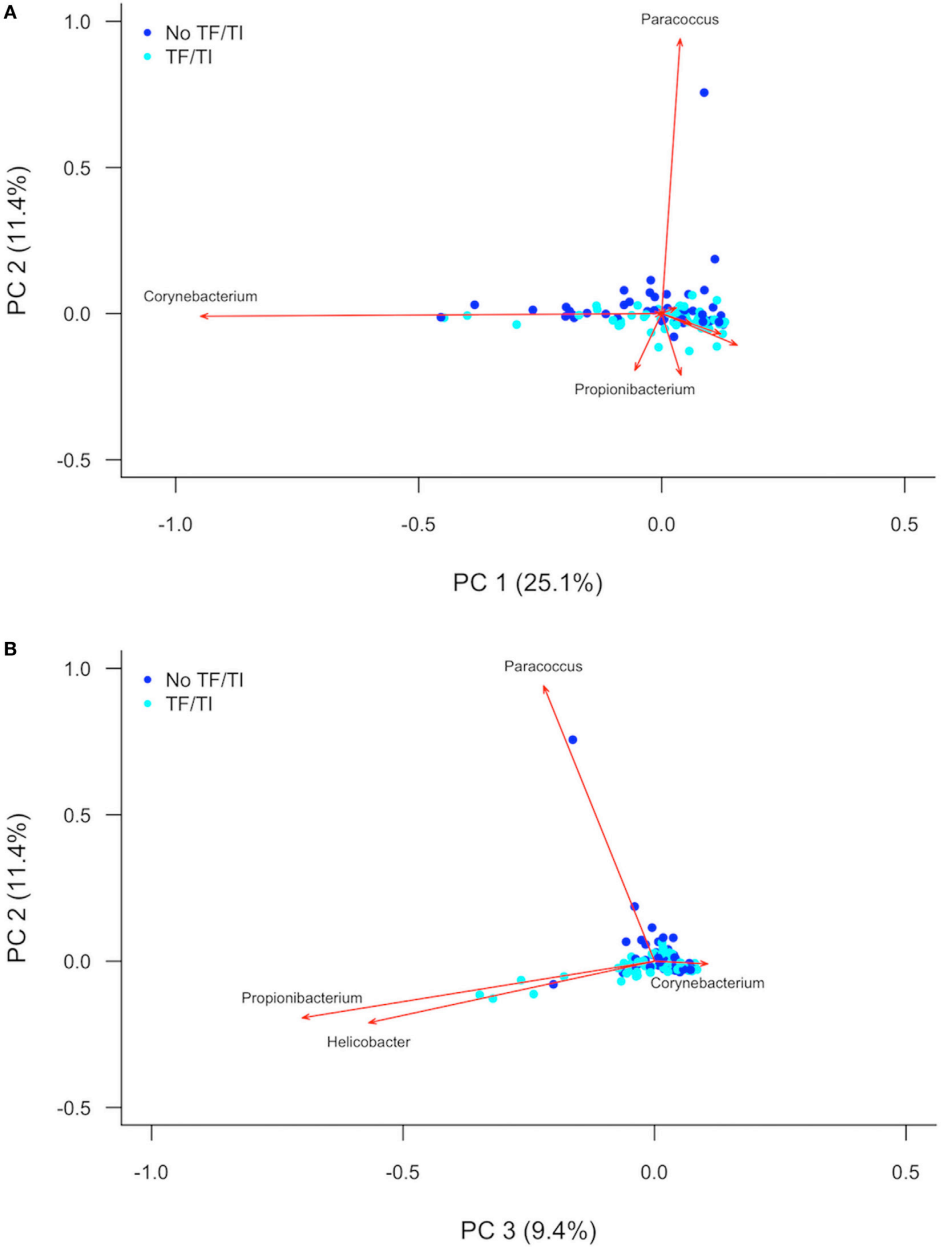
**(A)** First and second and **(B)** second and third principal components describing variation between the 16S sequences identified in Solomon Island children with and without active trachoma. Dark blue spots indicate controls. Light blue spots indicate cases. Red arrows show principal component loadings. PC, principal component; TF, trachomatous inflammation–follicular; TI, trachomatous inflammation—intense.

**Figure 3:**
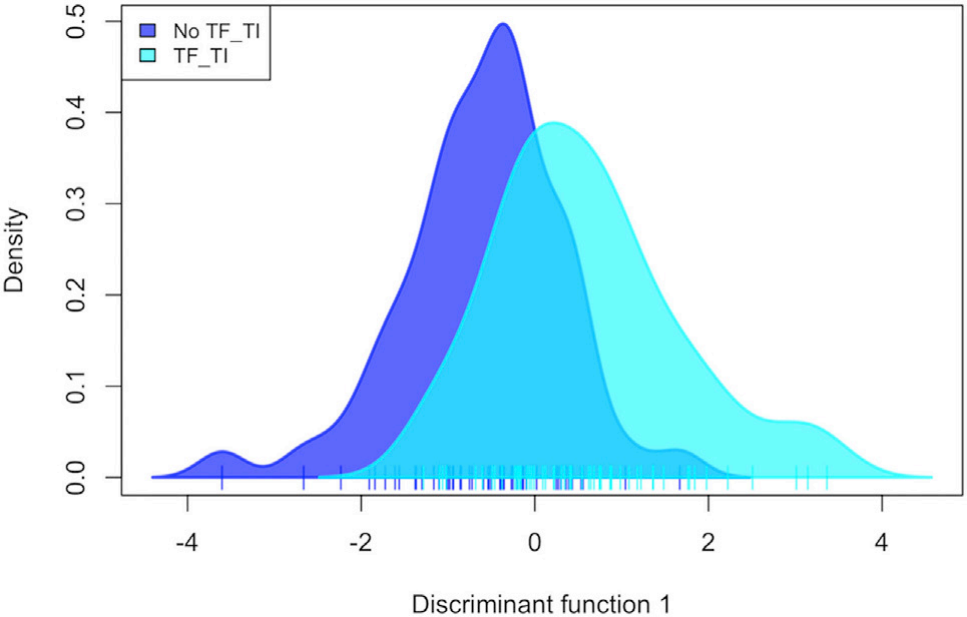
Discriminant analysis of the association of 20 combined principal components with active trachoma, showing a significant discrimination of phenotype groups (*p* = 0.000006). TF, trachomatous inflammation–follicular; TI, trachomatous inflammation—intense.

PC analysis (Figure 2; Figure S2 in Supplementary Material) revealed that *Corynebacterium* (PC1), *Paracoccus* (PC2), *Propionibacterium* (PC2 and PC3), and *Helicobacter* (PC3) are major contributors to the variation in the conjunctival microbiomes of children in the Solomon Islands. While individual cases or controls have distinctive profiles dominated by these genera, the majority of cases and controls are indistinguishable (Figure 2) using the first three PCs (explaining 45% of total variation). We condensed PCs 1–20 into a single discriminant function, which explained 87% of variation in the data. In this analysis, the genus level polymicrobial community structure is significantly different between cases and controls (logistic regression *p* = 0.0000062; Figure 3). That discriminant function was dominated by a number of genera such as *Curvibacterium* and *Mesoplasma* (Figure S3 in Supplementary Material). Cross-validation to predict group membership using discriminant functions of between 10 and 90 PCs had a low success rate (median 53.3% success; range: 50.1–62.3%). This suggests that although differences do exist between the polymicrobial communities in cases and controls, they are too subtle and varied to be predictive of phenotype, at least when working with this number of specimens.

## Discussion

Follicular conjunctivitis meeting criteria for the trachoma phenotype TF is highly prevalent in the Solomon Islands, but the prevalence of *Ct* infection is curiously low. Although *Ct* was the only pathogen to associate with TF in this study, the prevalence was much lower than may be expected of a population with this level of TF (3). Both *S. pneumoniae* and *H. influenzae* were detected at moderate prevalence in our population, while we found very few cases of *Adenoviridae* and *M. catarrhalis* infection. Comparator data from other populations are scarce, but it is clear that staphylococcal species were detected in the conjunctivae in the Solomon Islands at a substantially lower prevalence (7%) than in children in Tanzania (14.8% *S. epidermidis*) (7), Sierra Leone (20% *S. aureus* and 29% coagulase-negative *Staphylococcus*) (38), or The Gambia (post-MDA, 14.7% *S. aureus*) (11). By ddPCR testing for a number of bacteria and viruses that have previously been linked to TF, we can discount the possibility that any of them can account either singly or *en masse* for the high TF prevalence.

Through 16S amplicon sequencing and community profiling, we have shown that there is apparently no dominant bacterial genus associated with this disease. The dominant features of variation in conjunctival bacterial communities in Solomon Islands children (*Corynebacterium* and *Propionibacterium)* have consistently been identified in other studies. Other genera (e.g., *Paracoccus, Helicobacter, Haemophilus)* have not previously been identified as being important contributors to the conjunctival microbiome in 16S studies, while some reported in other studies (e.g., *Simonsiella, Pseudomonas*) were not found in these specimens (18–21). Many studies have sequenced the V3–4 region of the 16S gene, whereas we targeted V1–3. V region choice has been shown to have an impact on the genera identified at other mucosal sites (39), and while no data have yet emerged on how this affects profiles from the eye, it is possible this could account for some of the differences. Consistent with previous studies (36), we found a background of genera that amplified in NTCs that also appeared in clinical specimens. The microbiological biomass at the conjunctiva is known to be very low, even compared with other nearby sites such as skin or oral mucosa (19). When true resident bacteria are scarce, 16S rRNA gene PCR readily amplifies reagent contaminants. We took stringent measures to exclude reagent contaminants, but this resulted in the removal of some important genera such as *Staphylococcus* and *Streptococcus*. Among the biggest contributors to between-conjunctiva variation were *Corynebacterium* and *Propionibacterium*, but these genera did not differentiate TF cases from controls (Figure 2). Previous investigations of the role of the conjunctival microbiome in ocular disease have also not shown significant differences. In The Gambia, there was an increased abundance of *Haemophilus* in cases of TF, compared to controls, although this difference was not significant (21). People with ocular manifestations of rosacea, Sjögren’s syndrome, and healthy controls did not have significant differences in diversity or relative abundance of key phyla identified across all three groups (23). However, other disease associations have been identified. For example, the abundance of *Corynebacterium* and *Streptococcus* varied significantly between individuals with and without conjunctival scarring (21).

The microbiota of these children were heterogeneous and had subtle variations that appeared to reach significant association with TF when the full community structure was considered in comparative statistical models. It remains possible (though perhaps unlikely) that a multitude of factors, operating at the level of single bacterial species, polymicrobial communities, or viral species, all contribute to the presentation of the phenotype. It is perhaps more likely that as yet unidentified viral or allergic causes could explain the prevalence of TF in the Solomon Islands.

There are some limitations to our study. First, although the ddPCR assays are based on the validated assays and appear to be accurate (Table S1 in Supplementary Material), they have not been formally evaluated in this format. Second, by not using mechanical lysis in our extraction process, the absolute prevalence of some difficult-to-lyse Gram-positive genera (e.g., *Staphylococcus*) may have been underestimated. This would not impair the comparison of cases to controls, between which protocols were consistent. Third, 16S amplicon sequencing of low biomass samples is known to result in amplification of reagent and environmental contaminants (36). This study focused on gross differences in community structure between those with and without disease, and these should be independent of contaminating reads. We also employed stringent quality control measures to ensure data were not confused by artifacts of the sequencing process.

Given the current international commitment to trachoma elimination, further characterization of the role of non-*Ct* stimuli in TF is important. We might expect those with TF in the absence of *Ct* to be at lower risk of progression to TS and TT than those with repeated *Ct* infection and inflammation (40); however, such individuals have not been studied longitudinally to assess outcomes, and therefore, it is unclear how these cases should be managed. Furthermore, years after MDA has been administered, TF persists at levels above the threshold for elimination (> 5%) in communities where *Ct* infection is not readily detectable (41, 42). The public health risk of trachoma in these communities is also unclear.

Public health scenarios such as the one in the Solomon Islands will become increasingly common as trachoma elimination programmes reduce the global prevalence of *Ct*, and the positive predictive value of TF for ocular *Ct* infection declines as a result (43). MDA might be inappropriately delivered when clinical signs of trachoma are the only indicator used for programmatic decision-making. While MDA is effective for the treatment of trachoma (44), mass antibiotic exposure may increase macrolide resistance in non-chlamydial bacteria (45) and theoretically could make the population more susceptible to later infections by preventing accumulation of acquired immunity to *Ct* (46). Therefore, decisions to undertake such a program should be carefully considered. The case for using tests for *Ct* infection during trachoma surveys is strengthened by our data. The value of nucleic acid amplification tests for detecting non-chlamydial infection remains questionable, as multiple infectious agents might be important, and these may differ between populations.

## Ethics Statement

A parent or guardian gave written informed consent for each child to take part in the survey in accordance with the Declaration of Helsinki. The protocol was approved by the London School of Hygiene & Tropical Medicine (6319/6360) and the Solomon Islands National Health Research (HRC13/18) Ethics Committees.

## Author Contributions

Conceived and designed the study: RB, OS, RM, AS, DM, and ChR. Performed the fieldwork: RB, OS, KJ, EK, LS, and CR. Performed the experiments: RB, MH, JH, CP, and ChR. Analyzed the data: RB and ChR. Wrote the manuscript: RB and ChR. Revised and approved the manuscript: RB, OS, KJ, EK, LS, CR, JH, CP, MH, RM, AS, DM, and ChR.

## Conflict of Interest Statement

The authors declare that the research was conducted in the absence of any commercial or financial relationships that could be construed as a potential conflict of interest.
