# Supplementary material for: Active Trachoma Cases in the Solomon Islands Have Varied Polymicrobial Community Structures but Do Not Associate with Individual Non-Chlamydial Pathogens of the Eye
Source: Front Med (Lausanne). 2018 Jan 23;4:251. doi: 10.3389/fmed.2017.00251 (PMC5787070; doi:10.3389/fmed.2017.00251)
Supplement: Supplementary file 1 [file Data_Sheet_1.DOCX]

**Active trachoma cases in the Solomon Islands have varied polymicrobial community structures but do not associate with individual non-chlamydial pathogens of the eye.**

Robert M R Butcher, Oliver Sokana, Kelvin Jack, Joanna Houghton, Christine Palmer, Martin J Holland, Richard T Le Mesurier, Anthony W Solomon, David C W Mabey, Chrissy h. Roberts.

**Supplementary information**

**Supplementary table 1:** Each PCR product for dilution into standards was prepared using TaqMan Universal II PCR mix (Life Technologies, Paisley, UK), cleaned with Qiagen MinElute PCR product kit (Qiagen, Manchester, UK) and serially diluted from 1:10^6^ to 1:10^12^ in ten-fold dilution steps. Each series was tested in five technical replicates with duplex ddPCR assays to determine the reproducibility of the assay. Target concentrations between one and 10 copies/μL were reproducibly detected by all six assays. The coefficient of determination for all assays was in excess of 0.99 when fitted to a linear regression model. All six assays were highly reproducible, and the mean coefficient of variance (CoV) was 12.1% (range: 7.8 – 14.7%).

| Assay | Standard curve * | | |
| --- | --- | --- | --- |
|  | LoD | R^2^ | CoV (%) |
| *S. aureus* | 4.0 | 0.991 | 14.4 |
| Coagulase-negative *Staphylococcus* | 1.2 | 0.994 | 14.7 |
| *S. pneumoniae* | 9.9 | 0.998 | 11.9 |
| *H. influenza* | 2.3 | 0.997 | 14.7 |
| *Adenoviridae* | 3.0 | 0.997 | 9.1 |
| *M. catarrhalis* | 1.0 | 0.993 | 7.8 |

CoV: Coefficient of variation; LoD: Limit of detection: R^2^: Coefficient of determination

* Values calculated from 10-fold dilution series of PCR product between approximately 10^5^ and 10^1^ copies per target per test with 5 technical replicates at each dilution point.

**Supplementary figure 1.** Relative taxa abundance in age-, sex- and location-matched children with TF/TI (n = 54) and without (n = 53). Relative taxa abundance is expressed as percentage of total reads per specimen group. Genera with reads representing less than 1% of the total number of reads were combined into a group entitled ‘Other’.


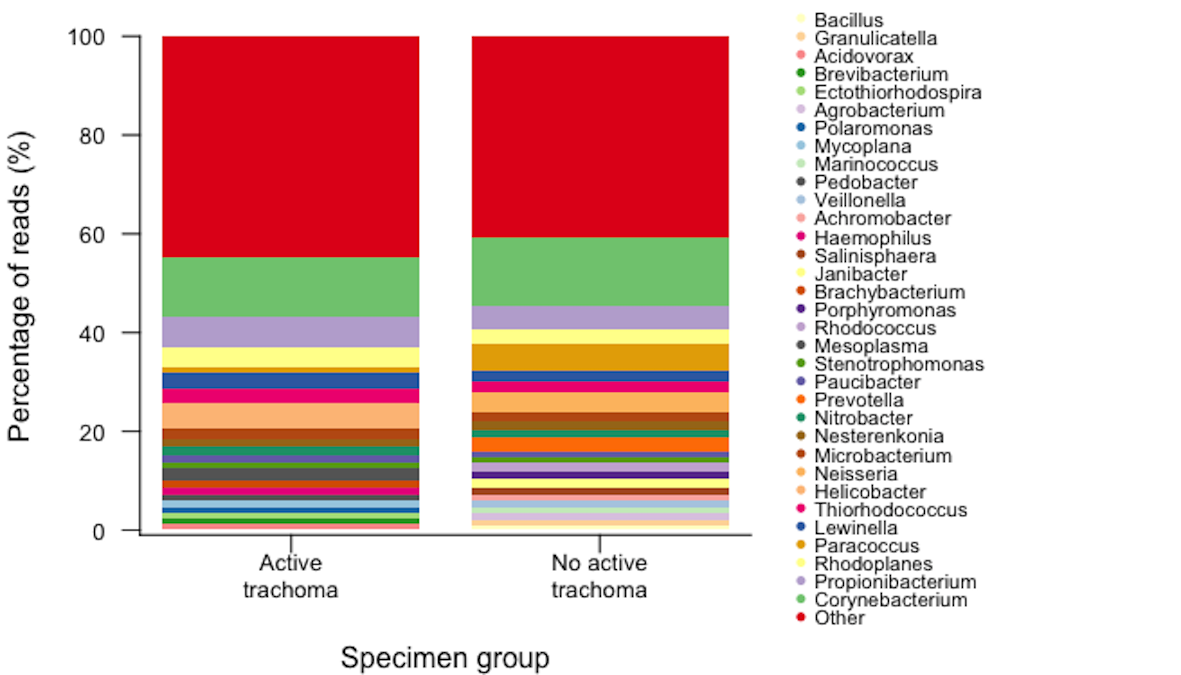


**Supplementary figure 2.** First and second principal components coloured by **(A)** age group and **(B)** gender. Spots indicate individuals. Red arrows indicate loadings.

**A**

**
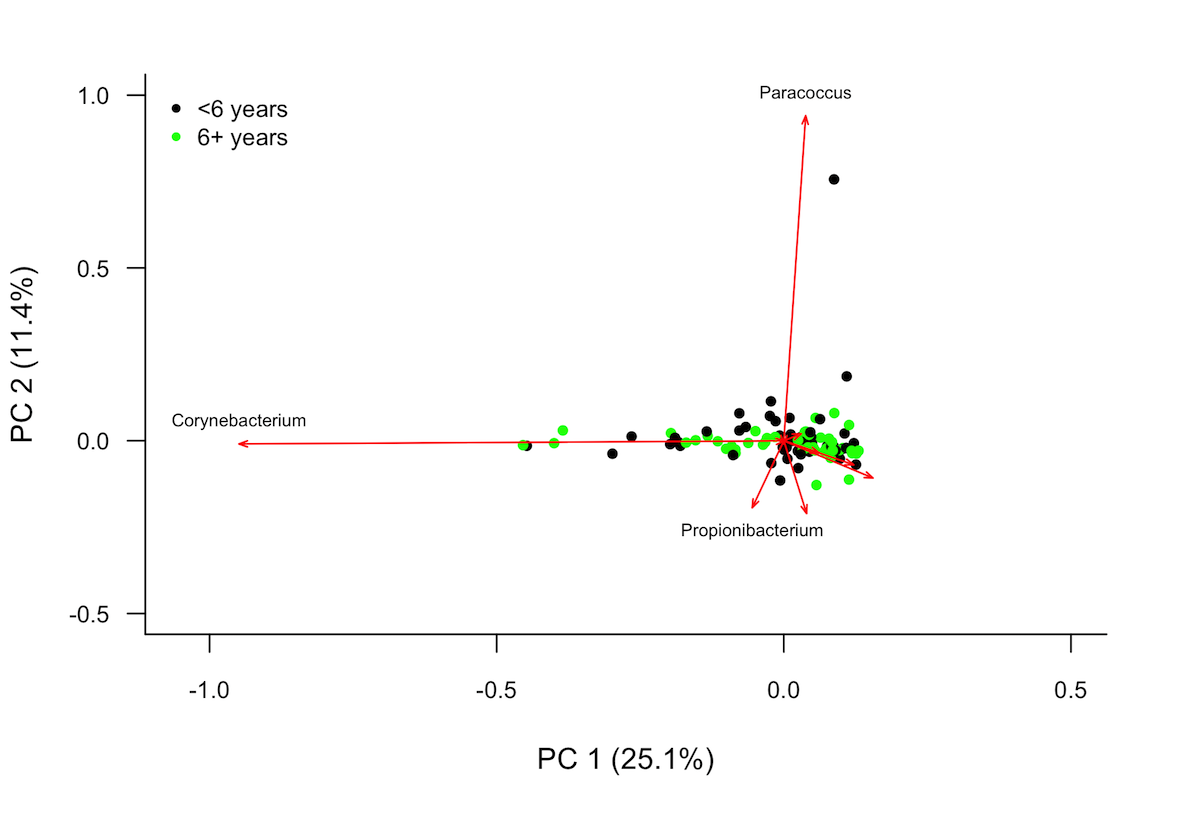
**

**B**

**
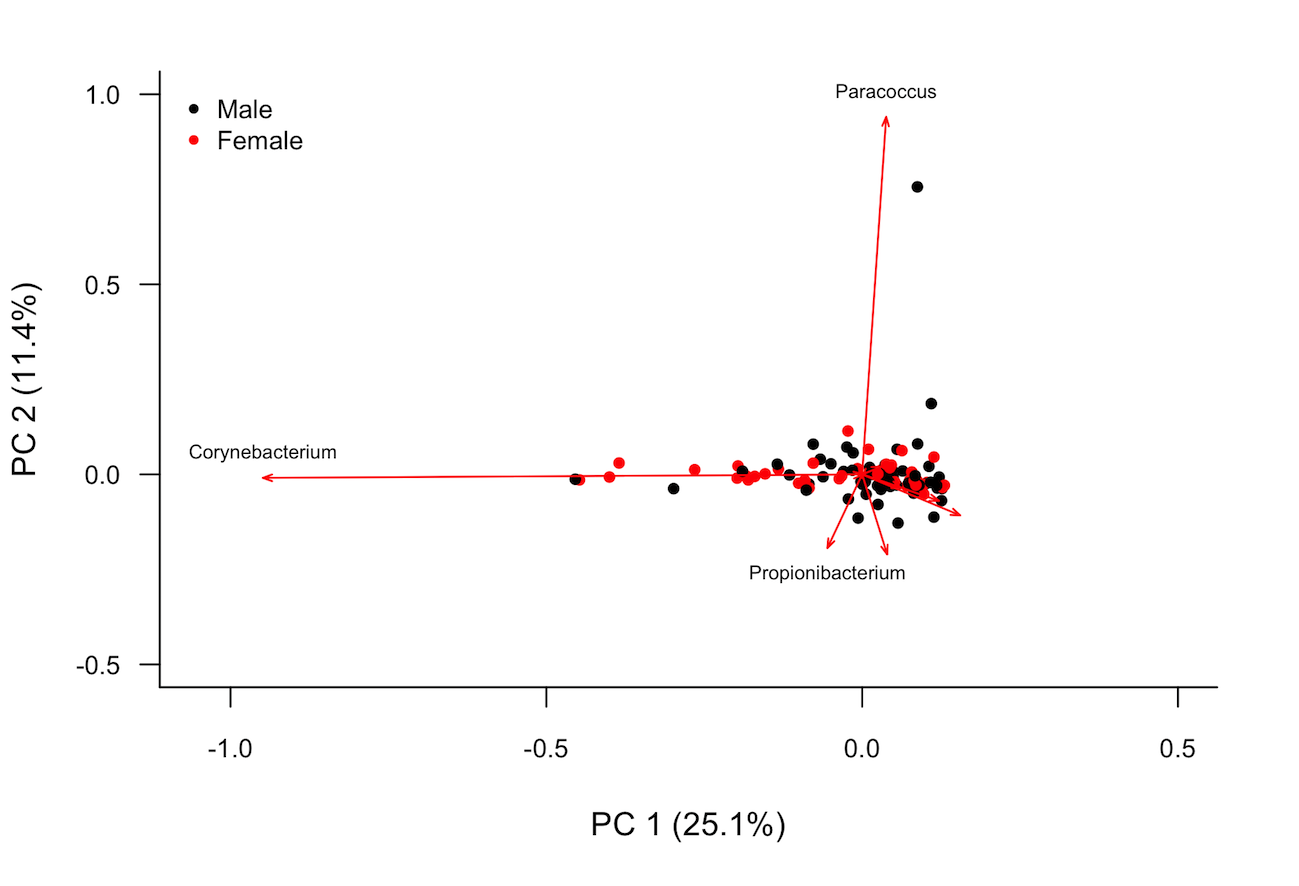
**

**
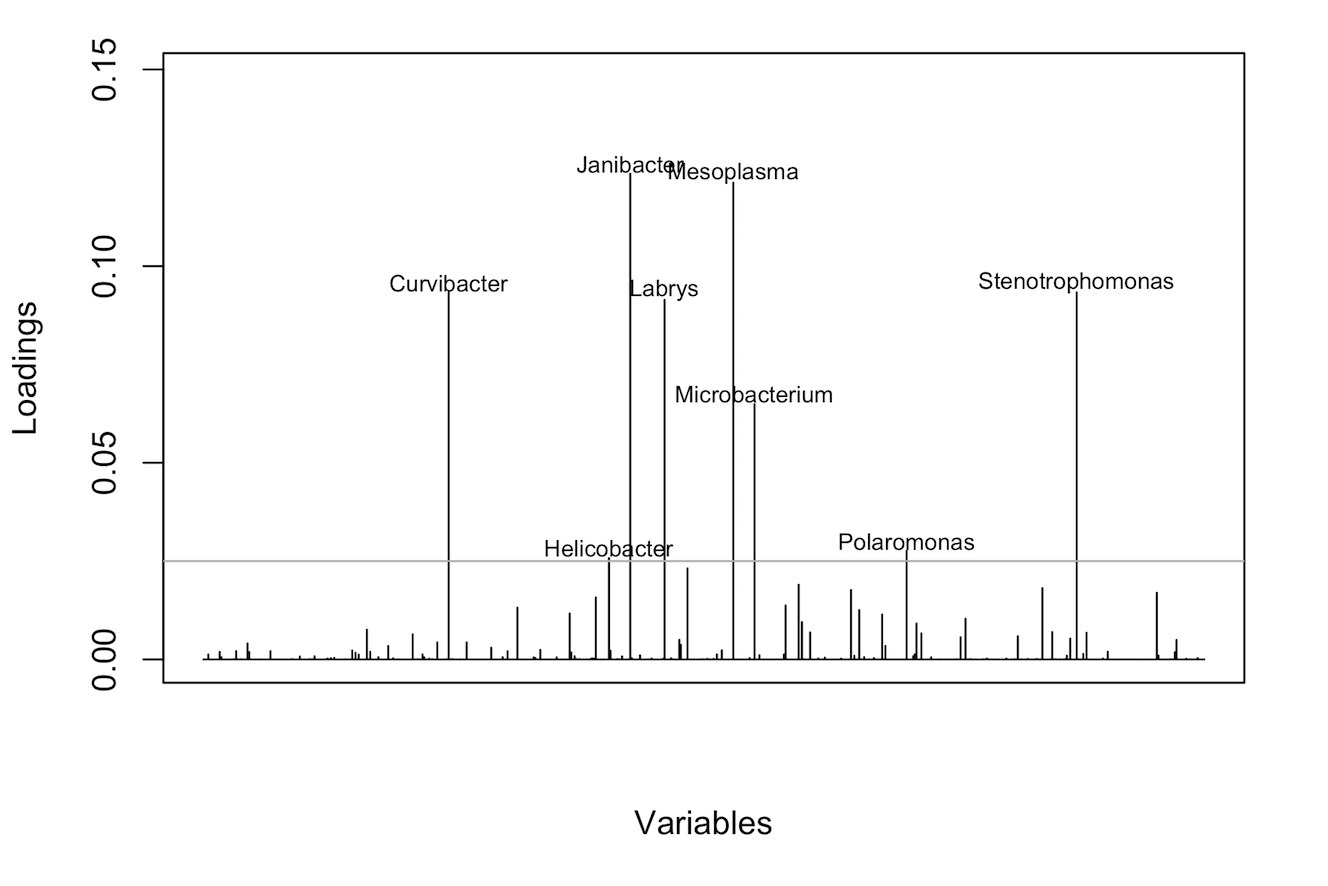
Supplementary figure 3.** Relative contributions of genera driving difference between active trachoma cases and controls
